# Supplementary material for: No effect of multi-axis dot pattern symmetry on subjective duration
Source: PLoS One. 2020 Dec 31;15(12):e0238554. doi: 10.1371/journal.pone.0238554 (PMC7775060; doi:10.1371/journal.pone.0238554)
Supplement: Attachment — Submitted filename: PONE-D-20-25416_Review1_WRoseboom.pdf [file pone.0238554.s001.pdf]

**Overall opinion**

Out of 5 stars rating (based on percentages below) ☆☆☆☆☆

Summary of paper (a few sentences):

| |
|---|
| Previous work suggests an effect of visual symmetry on estimates of duration. The present manuscript seeks to extend on these findings by manipulating the number of folds axis reflections) in the stimuli. The justification for using these kinds of stimuli is only briefly provided in terms of "perceptual goodness". The results suggest that not only is there no evidence for an influence of the new "folds" manipulation, but also the original symmetry bias of duration can't be reproduced. The paper in general is presented within an metascience argument about the value of presenting nulls to avoid file draw issues. |

Summary of opinion (a few sentences):

| |
|---|
| I think that it's good that the authors are actively seeking to reduce file drawer issues. I appreciate that they may be concerned that editors and reviewers may not appreciate this and so have presented the paper in a metascience context, largely. I think that more can be done to introduce the motivations that led to the study being run (why did you want to investigate number of folds to begin with?). Providing the materials and data is great. I would appreciate if more effort were made to make the data and analyses (see below) easy to interpret for a reader. There are a few places (both methodologically and conceptually) where I think the paper would benefit from more clarity/details. The one big issue I can see is that claims about being a failed replication or similar are hampered by being a *conceptual replication* without any evidence of a strong manipulation check. In the discussion the authors start in on addressing this issue, which is great, but only assert that the symmetry in the used stimuli is sufficient. I think that presenting some evidence to support this claim is required. Overall a brief and, other than the above issues, sound paper to my reading. |

**Person making opinion**

| | |
|---|---|
| Name: | Warrick Roseboom |
| Affiliation: | University of Sussex |
| Relationship to authors: | None |

Are you an expert on this topic/method? Please specify aspects of this study that you feel you are expert in and those which you are not (e.g. you know a lot about the topic, but maybe not so much about the precise methods used here, or vice versa):

| |
|---|
| I would consider myself relatively expert in both the topics and methods used in this manuscript. I work on models of human time perception and frequently use human participants in tasks with designs and analyses similar to those used here. I re-read the Ogden et al. 2016 paper in preparation. |

Consult also the following for alternative/related checklists:

10.1038/s41562-019-0772-6

10.31234/osf.io/edh2s

10.1177/2515245918806489

**Transparency (30/50)**

| | |
|---|---|
| 1. Are the data presented in the paper available on a public repository? (make sure this is noted in "availability statement" or similar) | yes/no/comment |
| The data are available at a linked osf project site, which is great. The data are in a .xlsx file, with the different data (along with summary and figures) for each experiment on different tabs of the file. Correct me if I'm wrong, but these appear to be the only the cell-wise averages? It would be helpful if there were more notes/comments about what the presented data are etc, so that someone grabbing the data can immediately understand what they are looking at without any guesswork. If it is indeed the cell-wise summary, it would be better it the raw trial by trial data were also included. | |
| 2. Is the code to reproduce the results available with the data? (make sure this is noted in "availability statement" or similar) | yes/no/comment |
| As above, the analyses to produce summary statistics are present on the .xlsx file, but the relation to the raw trial by trial data is not present.<br>I additionally note that the authors have provided the materials for running the experiment. I didn't check whether these work or are accurate to the provided description. | |
| 3. Have you checked the code/data? | yes/no/NA |
| 4. Does the code run without requiring unreasonable amendments? (e.g. changing directories, downloading repositories, fixing versioning is fine. Broken code or code not producing same results not fine). | yes/no/NA |
| 5. Does the paper use inferential statistics? (e.g. any statistical test/model/method against a criterion) | yes/no |
| 6. Is there a justification provided for the sample size in relation to this test(s)? | yes/no/NA |
| 7. If yes, is the provided justification reasonable? (because "someone used this sample before" is not reasonable) | yes/no/comment |
| There is a justification based on partial-eta-square. This is good. I would like to see a more thorough explanation of the process and justification here. Daniel Lakens had a nice blog post (and follow up paper) several years ago about how no one talks about power and so no one | |

| | |
|---|---|
| knows about it. It would be great to provide more details (and include materials on osf) that support the provided justification. | |
| 8. Are the primary hypotheses/analysis methods pre-registered? | yes/no |
| 9. Does the preregistration robustly reduce researcher degrees of freedom? (ideally, for pre-registered tests there should be no discernable researcher dof) | yes/no/NA |
| 10. Do the presented analyses deviate from the preregistration in any way? (if yes, please identify deviations) | yes/no/NA |
| 11. If yes, are the deviations justified/reasonable? | yes/no/comment |
| - | |
| 12. If present, are exploratory analyses clearly marked as such? | yes/no/NA |

**Content (31/50)**

**Introduction (3/5)**

| | |
|---|---|
| 13. Are the presented hypotheses well-motivated by presented literature? | yes/no/comment |
| In a very brief way, yes. I understand the motivation to present the paper in view of a metascience position, potentially for fear that a reviewer would reject on the basis of not being novel. Given the context and the place it has been submitted, I would consider providing more background on the original motivation of the study. Little is given in way of justification for looking at number of folds and it seems this was the original motivating factor. I understand that this kind of comes to nothing given the results, but given that it seems you set out to investigate this (rather than looking to disconfirm the previous results) some background motivating your thinking is probably warranted. | |
| 14. Are the presented hypotheses based on theories or assumptions that are reasonable or supported by the literature?[1] | yes/no/comment |
| The presented hypothesis is quite narrow (perhaps reflecting my comment from directly above). The details relating both to time and gestalt are a little murky. The "arousal" idea for internal clocks is just a description of the results, in the literature (including the cited literature) always an ad hoc interpretation. Maybe again reflecting that the motivations aren't made entirely clear for this specific study, but the idea that these particular stimuli are going to be | |

[1] For example, hypotheses that are based on homuncular accounts of cognition (e.g. "we track time using an internal stopwatch, therefore if X then Y") are unreasonable. It is also common to misinterpret measures as theories, e.g. intentional binding is an implicit measure of sense of agency, therefore when I measure temporal binding I am measuring implicit agency. Are any caveats/limitations like this acknowledged, either in the introduction or the discussion?

| | |
|---|---|
| useful for this case because of their “perceptual goodness” (bottom of p.5) is not clearly justified to my reading. | |
| 15. Is the presented literature review a fair reflection of the existing literature?[2] | yes/no/comment |
| In so far as the metascience issues, I think so. As above, I think it would be appropriate to balance this discussion with the motivation for the study further. But what is there is fair. | |
| 16. Is previous work cited appropriately?[3] | yes/no/comment |
| As far as I can tell. I didn’t follow through with every reference but didn’t see anything inappropriate. | |

**Results (25/35)**

| | |
|---|---|
| 17. Have the analysis methods been applied correctly (as specified) as far as you can tell?[4] | yes/no/comment |
| As mentioned above, it would be good to have more clarity on how power was calculated.<br><br>Is it necessary to conduct both Shapiro-Wilk and K-S test? K-S test is potentially problematic for empirical distributions that are not *necessarily* normal (when comparing with normal) and is problematic (generally unreliable) for samples of this size.<br><br>A quick glance at the Fs and associated p-values seems correct but I didn’t explicitly check whether these were correct. | |
| 18. Is the paradigm appropriate for testing the hypothesis?[5] | yes/no/comment |
| Again, as the hypothesis appears to be quite limited – is there evidence for an effect of “folds” on duration judgements. With this very limited question, then yes. How this extends to the other questions presented in the previous papers, I guess the authors are deliberately staying | |

[2] Note, this doesn’t mean comprehensive. Not all papers need to be review papers. Make sure to provide full references to papers you believe to be relevant but missing.
[3] Cited work not relevant, too general, misinterpreted, retracted, unreliable or debunked, citing secondary sources, overuse of review papers. If no, provide explanation.
[4] e.g. no optional stopping in frequentist test; no impossible degrees of freedom/t values/p values. See Appendix A in doi:10.1177/2515245918806489 for comprehensive list of things to look out for.
[5] e.g. when making claims about how participants’ perception of a stimulus differs between conditions, does the task measure performance (sensitivity) or just propensity to report (bias); if you don’t know what this means refer to Kingdom and Prins, Psychophysics: A Practical Introduction, Chapter 2. Another common problem is the experimental manipulation not being precise enough, e.g. assuming that TMS can selectively target particular cells; TMS to the neck would not be an appropriate way of testing the causal role of baroreceptors in interoception, because TMS would stimulate the whole neck, not just baroreceptors.

| | |
|---|---|
| away from this. | |
| 19. Are there any clear methodological confounds/errors in the results? (provide a full explanation of why, citing references where necessary). | yes/no/comment |
| I guess there is an issue conceptually with concluding in favour of the null in any substantive, broader way here because it is effectively a conceptual replication. Perhaps it is trivial in this instance, but there is no explicit manipulation check (on random vs reflection) presented here. There is also no contrast with the previously used stimuli (e.g. Ogden et al. 2016). Sure, this might be trivial in that they are very easy for participants to detect which one is the symmetrical one and which is not, and the salience of the symmetry is similar to previous studies, but there is no evidence presented for this which makes any stronger conclusion tricky (related to comments on discussion below). I think to make strong comment about whether this is indeed a failed replication or simply doing something slightly different (which reflects on the broader notion of visual symmetry) that this kind of information is necessary. | |
| 20. Are there any analyses of the presented data that you can think of that have not been conducted and would potentially reflect alternative interpretations?<br>(provide a full description of the additional analyses required, citing references where necessary, along with explicit prediction of the results, and explanation of why these results are expected). | yes/no/comment |
| As above, very tricky given the "null" result. But the presented evidence is very straightforward. The larger concerns would be about manipulation checks. | |
| 21. Do the data figures make sense and have clear labelling and explanation? (give suggestions of how the figures could be made more intuitive for you). | yes/no/comment |
| | |

**Discussion (3/5)**

| | |
|---|---|
| 22. Does the Discussion provide a fair natural language account of what happened in the experiments in relation to stated hypotheses? | yes/no/comment |
| Very brief, though fair. First paragraph of the discussion makes a simple overall statement that there was no evidence for either of the key manipulations having any effect. | |
| 23. Does the Discussion provide a fair account of the links between the obtained results and motivating results outlined in the Introduction? | yes/no/comment |
| I think again here it is difficult. The authors acknowledge that the stimuli used here are different from previous studies, but then assert that they are more salient (second paragraph, page 11). As above, I'm generally happy to accept this is true, but a stronger statement would | |

| |
|---|
| be to show evidence that these are as salient, or at least “sufficiently” salient, whatever that might mean. |

| | |
|---|---|
| 24. Are there any other relevant relationships between the results observed in this study and other literature that have been overlooked by the authors?[6] | yes/no/comment |
| Nothing that comes to mind immediately. Again, the contrast conducted is very simple and there is no evidence that the manipulations (other than duration) have any effect. So the only thing to again mention is about symmetry in general and whether the manipulation was effective.<br>Breaking the rules of the template slightly here but possibly relevant to the issue of whether changing the report task is this paper https://psyarxiv.com/pg7bs/. This is not a request for citation, just a note related to the fact that you seem to be trying to defend that the analog scale versus verbal estimation wouldn’t necessarily be a problem. | |

**Wildcard (0/5)**

| | |
|---|---|
| Does the paper develop/deploy a method in a new and/or interesting way? | yes/no/comment |
| | |
| Does the paper provide a link between known results in an interesting way? | yes/no/comment |
| | |
| Is there generally something about this paper that makes you go “Wow, I wish I had thought of that”? | yes/no/comment |
| | |

**Minor comments**

Please identify basic mistakes that you notice like typos, grammatical errors, or sections of text that could otherwise be expressed more clearly. Provide suggested alternative phrasing if you wish.

| |
|---|
| 1. On page 7 when describing the stimulus “The SD of dots…”. Consider not using abbreviation for clarity. |

[6] Note, this is not meant for you to request references to your own work unless actually related.

2. Page 8. “… so we effectively continuous.”)
