# Supplementary material for: No effect of multi-axis dot pattern symmetry on subjective duration
Source: PLoS One. 2020 Dec 31;15(12):e0238554. doi: 10.1371/journal.pone.0238554 (PMC7775060; doi:10.1371/journal.pone.0238554)
Supplement: Attachment — Submitted filename: Response to reviewers V2.docx [file pone.0238554.s002.docx]

Please find our responses below (highlighted yellow)

PONE-D-20-25416
A failure to replicate the effect of visual symmetry on subjective duration
PLOS ONE

Dear Dr. Makin,

Thank you for submitting your manuscript to PLOS ONE. After careful consideration, we feel that it has merit but does not fully meet PLOS ONE’s publication criteria as it currently stands. Therefore, we invite you to submit a revised version of the manuscript that addresses the points raised during the review process.

Please submit your revised manuscript by Dec 26 2020 11:59PM. If you will need more time than this to complete your revisions, please reply to this message or contact the journal office at plosone@plos.org. When you're ready to submit your revision, log on to https://www.editorialmanager.com/pone/ and select the 'Submissions Needing Revision' folder to locate your manuscript file.

Please include the following items when submitting your revised manuscript:

- A rebuttal letter that responds to each point raised by the academic editor and reviewer(s). You should upload this letter as a separate file labeled 'Response to Reviewers'.
- A marked-up copy of your manuscript that highlights changes made to the original version. You should upload this as a separate file labeled 'Revised Manuscript with Track Changes'.
- An unmarked version of your revised paper without tracked changes. You should upload this as a separate file labeled 'Manuscript'.

If you would like to make changes to your financial disclosure, please include your updated statement in your cover letter. Guidelines for resubmitting your figure files are available below the reviewer comments at the end of this letter.

If applicable, we recommend that you deposit your laboratory protocols in protocols.io to enhance the reproducibility of your results. Protocols.io assigns your protocol its own identifier (DOI) so that it can be cited independently in the future. For instructions see: <http://journals.plos.org/plosone/s/submission-guidelines#loc-laboratory-protocols>

We look forward to receiving your revised manuscript.

Kind regards,

Guido Hesselmann
Academic Editor
PLOS ONE

Journal Requirements:

When submitting your revision, we need you to address these additional requirements.

1. Please ensure that your manuscript meets PLOS ONE's style requirements, including those for file naming. The PLOS ONE style templates can be found at

https://journals.plos.org/plosone/s/file?id=wjVg/PLOSOne_formatting_sample_main_body.pdf and

https://journals.plos.org/plosone/s/file?id=ba62/PLOSOne_formatting_sample_title_authors_affiliations.pdf

2. PLOS ONE does not copy edit accepted manuscripts. Please proofread for typos and grammar. In addition, please change "female” or "male" to "woman” or "man" as appropriate, when used as a noun."

3. Please provide additional details regarding participant consent. In the ethics statement in the Methods and online submission information, please ensure that you have specified (1) whether consent was informed and (2) what type you obtained (for instance, written or verbal, and if verbal, how it was documented and witnessed). If your study included minors, state whether you obtained consent from parents or guardians. If the need for consent was waived by the ethics committee, please include this information.

We have now altered the ethics statement.

If you are reporting a retrospective study of medical records or archived samples, please ensure that you have discussed whether all data were fully anonymized before you accessed them and/or whether the IRB or ethics committee waived the requirement for informed consent. If patients provided informed written consent to have data from their medical records used in research, please include this information.
Once you have amended this/these statement(s) in the Methods section of the manuscript, please add the same text to the “Ethics Statement” field of the submission form (via “Edit Submission”).

For additional information about PLOS ONE ethical requirements for human subjects research, please refer to http://journals.plos.org/plosone/s/submission-guidelines#loc-human-subjects-research.

4. Thank you for stating in your Funding Statement:

"This work was partly funded by the Economic and Social Research Council UK award to AM

https://esrc.ukri.org/

ES/S014691/1

The funders had no role in study design, data collection and analysis, decision to publish, or preparation of the manuscript."

Please provide an amended statement that declares *all* the funding or sources of support (whether external or internal to your organization) received during this study, as detailed online in our guide for authors at [http://journals.plos.org/plosone/s/submit-now](about:blank).  Please also include the statement “There was no additional external funding received for this study.” in your updated Funding Statement.

Please include your amended Funding Statement within your cover letter. We will change the online submission form on your behalf.

Thank you for giving us an opportunity to resubmit this manuscript to PLOS One.

We have removed ‘partly’ from the funding statement.

Our response to reviewers is included below. Changes to the new manuscript are highlighted yellow.

Reviewers' comments:

Reviewer's Responses to Questions

**Comments to the Author**


Previous work suggests an effect of visual symmetry on estimates of duration. The present manuscript seeks to extend on these findings by manipulating the number of folds axis reflections) in the stimuli. The justification for using these kinds of stimuli is only briefly provided in terms of “perceptual goodness”. The results suggest that not only is there no evidence for an influence of the new “folds” manipulation, but also the original symmetry bias of duration can’t be reproduced. The paper in general is presented within an metascience argument about the value of presenting nulls to avoid file draw issues.

This is a very accurate summary of the paper. We did decide to focus largely on the meta science after seeing the null result, but this probably went too far.

We have copied substantial comments from the proforma peer review and addressed them here. See yellow highlights for responses.

I think that it’s good that the authors are actively seeking to reduce file drawer issues. I appreciate that they may be concerned that editors and reviewers may not appreciate this and so have presented the paper in a metascience context, largely. I think that more can be done to introduce the motivations that led to the study being run (why did you want to investigate number of folds to begin with?). Providing the materials and data is great. I would appreciate if more effort were made to make the data and analyses (see below) easy to interpret for a reader. There are a few places (both methodologically and conceptually) where I think the paper would benefit from more clarity/details. The one big issue I can see is that claims about being a failed replication or similar are hampered by being a conceptual replication without any evidence of a strong manipulation check. In the discussion the authors start in on addressing this issue, which is great, but only assert that the symmetry in the used stimuli is sufficient. I think that presenting some evidence to support this claim is required. Overall a brief and, other than the above issues, sound paper to my reading.

We are pleased reviewer 1 feels this is a sound paper and approves of our response to the file drawer problem. It is true that this is a failed conceptual replication, not a failed exact replication. We have now changed out discussion to be much clearer about this important distinction, as described below.

The data are available at a linked osf project site, which is great. The data are in a .xlsx file, with the different data (along with summary and figures) for each experiment on different tabs of the file. Correct me if I’m wrong, but these appear to be the only the cell-wise averages? It would be helpful if there were more notes/comments about what the presented data are etc, so that someone grabbing the data can immediately understand what they are looking at without any guesswork. If it is indeed the cell-wise summary, it would be better it the raw trial by trial data were also included.

As above, the analyses to produce summary statistics are present on the .xlsx file, but the relation to the raw trial by trial data is not present.

I additionally note that the authors have provided the materials for running the experiment. I didn’t check whether these work or are accurate to the provided description.

The full raw trial data set was there on OSF, it was just in the *Experiment folder* rather than in the *Results folder*. We have now made a new ‘Raw behavioural data’ folder on OSF.

There is a justification based on partial-eta-square. This is good. I would like to see a more thorough explanation of the process and justification here. Daniel Lakens had a nice blog post (and follow up paper) several years ago about how no one talks about power and so no one knows about it. It would be great to provide more details (and include materials on osf) that support the provided justification.

We have expanded our section on power analysis with reference to Lakens (2013):

*“Power analysis*

*Power analysis is a recurring topic in the contemporary meta-science literature. Bishop (2019) lists low statistical power as one of the ‘four horsemen of irreproducibility’. Brysbaert (2019) suggests that psychologists routinely misunderstand power analysis, and many research programs are built on an unstable foundation of underpowered experiments (see also Lakens, 2013). A fundamental problem is that meaningful power analysis requires a reliable estimate of effect size. Brysbaert (2019) claims that ‘you need an estimate of effect size to get started, and it is very difficult to get a useful estimate” (page 7). We have just two relevant a priori estimates of effect size at our disposal (η_p_^2^ = 0.194 from Palumbo, Ogden et al. (2015) and 0.451 from Ogden et al. (2016)). Converting to Cohen’s d_z_ gives us 0.491 and 0.906. A conservative approach is to use the smaller of the two for power analysis.*

*With this smaller effect size of η_p_^2^ = 0.194 (d_z_ = 0.491) observed power was just 0.616. If this is the true population effect size, we have an unacceptably high chance of missing the effect by using the sample of 24 (p = 1-0.616 = 0.384). However, with 40 participants, we have an 0.85 chance of finding the effect. The required sample size for finding a modulatory effect of folds is unclear because we do not have an a priori estimate of effect size here.”*

We are not sure what would go on open science framework to supplement the information given in these paragraphs.

I understand the motivation to present the paper in view of a metascience position, potentially for fear that a reviewer would reject on the basis of not being novel. Given the context and the place it has been submitted, I would consider providing more background on the original motivation of the study. Little is given in way of justification for looking at number of folds and it seems this was the original motivating factor. I understand that this kind of comes to nothing given the results, but given that it seems you set out to investigate this (rather than looking to disconfirm the previous results) some background motivating your thinking is probably warranted.

It is true that we wrote the paper as an exercise in metascience *after seeing the null results*, and thus neglected the ‘original’ introduction. We have now expanded on this:

*“Research on symmetry and duration can only progress beyond these initial observations if we establish some basic parameters of the effect. For instance, does the effect scale of symmetry on duration with salience of symmetry, or is it an all or nothing phenomenon? The most reliable way to manipulate symmetry salience is to vary the number of folds, we began with this.”*

The presented hypothesis is quite narrow (perhaps reflecting my comment from directly above). The details relating both to time and gestalt are a little murky. The “arousal” idea for internal clocks is just a description of the results, in the literature (including the cited literature) always an ad hoc interpretation. Maybe again reflecting that the motivations aren’t made entirely clear for this specific study, but the idea that these particular stimuli are going to be useful for this case because of their “perceptual goodness” (bottom of p.5) is not clearly justified to my reading.

We have removed the brief reference to arousal. We were never strongly committed to claims about arousal and internal clock speed, although this was prominent in Ogden et al. (2016). We have made this more explicit, as we continue with the expanded introduction:

*“*If established, this could be a useful clue for understanding how duration estimation works at a neural level, which remains uncertain despite decades of research (Roseboom et al., 2019; van Rijn, Gu, & Meck, 2014; Wearden, 2013). Duration estimates could be mediated by an internal pacemaker-accumulator clock (Church, 1984; Wearden, Edwards, Fakhri, & Percival, 1998). However the internal clock model is controversial (Coull, Cheng, & Meck, 2011; Johnston, Arnold, & Nishida, 2006) and radically different alternatives have been proposed (Roseboom et al., 2019). New empirical evidence from exploratory studies like ours could help constrain future theories of time perception.

Example stimuli with 1-5 folds are shown in Figure 1. These particular dot patterns were chosen because they were used in Event Related Potential (ERP) research by Makin et al. (2016) and again in preference research by Makin, Helmy and Bertamini (2018). Using the same stimuli across research programmes can sometimes facilitate discovery of new connexions which would otherwise be missed. For instance, there could be an intriguing link between the amplitude of the neural response and subjective duration, suggesting time is coded as sensory excitation. By using consistent stimuli, such links are more likely to be revealed*.”*

As mentioned above, it would be good to have more clarity on how power was calculated. Is it necessary to conduct both Shapiro-Wilk and K-S test? K-S test is potentially problematic for empirical distributions that are not necessarily normal (when comparing with normal) and is problematic (generally unreliable) for samples of this size. A quick glance at the Fs and associated p-values seems correct but I didn’t explicitly check whether these were correct.

We agree it is probably not necessary to report both normality tests. We have therefore removed reference to K-S test.

I guess there is an issue conceptually with concluding in favour of the null in any substantive, broader way here because it is effectively a conceptual replication. Perhaps it is trivial in this instance, but there is no explicit manipulation check (on random vs reflection) presented here. There is also no contrast with the previously used stimuli (e.g. Ogden et al. 2016). Sure, this might be trivial in that they are very easy for participants to detect which one is the symmetrical one and which is not, and the salience of the symmetry is similar to previous studies, but there is no evidence presented for this which makes any stronger conclusion tricky (related to comments on discussion below). I think to make strong comment about whether this is indeed a failed replication or simply doing something slightly different (which reflects on the broader notion of visual symmetry) that this kind of information is necessary.

This is an important point which was underdeveloped in the original manuscript. As the reviewer says, there is a continuum between pure failure to replicate (two identical experiment do not show the same effect) and failure to *conceptually replicate,* which may sometimes be a genuine discovery of boundary conditions (the original effect is abolished by some minor variations in task and stimuli, originally assumed to be trivial). What we report here is a failed conceptual replication.

We have changed the title of the paper:

*“No effect of multi-axis dot pattern symmetry on subjective duration.”*

We have also added an important clarification at the end of the abstract:

*“ One possibility is that the effect of symmetry on subjective duration is limited to very specific experimental paradigms.”*

We have also made the important distinction explicit in the discussion:

*“It should be emphasised that what we report here is a failed conceptual replication (see Nosek & Errington, 2020 for careful consideration of different replication types).”*

As you say, a failed conceptual replication is inherently mysterious without further research. We can only speculate on plausible boundary conditions. We have now added three new paragraphs to the discussion, which expand on an existing one:

*“Our dot patterns were different from the checkerboards in Palumbo, Ogden et al. (2015) and Ogden et al. (2016) in a number of ways. For instance, they were more complex. Complexity can modulate perceived duration (Palumbo, Ogden, Makin, & Bertamini, 2014), so this could be part of the story. However, is unclear why increasing complexity should supress the difference between reflection and random conditions. Likewise, our stimuli were 5° diameter discs instead of 10° squares, but there is no obvious reason why this size change would abolish effect of symmetry on duration either.*

*Previous studies used durations ranging up to 1.6 seconds, while we used durations <1 second. Could this explain the inconsistencies with previous work? Ogden et al (2016) found the symmetry effect only for durations of less than a second, so it may be necessary to present a range of durations for the effect of symmetry to manifest at the lower end. Indeed, range effects are well known in the timing literature: Vierordt’s law states duration estimates tend to cluster round the mean presented (so the shorter durations are overestimated, and longer durations underestimated). Vierordt’s law could perhaps contribute to a multifaceted explanation for our null result.*

*A failed conceptual replication could also be uninteresting if the task had changed in an obviously problematic way. We cannot identify an obvious task-based explanation either. Palumbo, Ogden et al. (2015) and Ogden et al. (2016) used a verbal estimation task, where participants entered judgements using the keyboard. In contrast, our participants entered judgments using the mouse and continuous scale. Our new response entry method may have reduced ‘quantizing’ (where participants tend to give round number estimates such as “500 ms” or “1000 ms” Wearden, 2015). The extra precision could theoretically introduce ceiling effects that mask the effect of symmetry. However, the effect of objective duration on subjective duration was comparable to previous work (Current study η_p_^2^ = 0.85, Ogden et al. 2016, η_p_^2^ = 0.88, Palumbo, Ogden et al. 2015, η_p_^2^ = 0.76). This suggests that suppressive ceiling effects were no greater in the current experiment.*

*The above paragraphs demonstrate how failed conceptual replications can arise from previously unknown boundary conditions. Some change in stimulus or task, originally assumed to be trivial, has the unforeseen consequence of abolishing the expected effect. Palumbo, Ogden et al. (2015) and Ogden et al. (2016) might have been lucky by choosing parameters that worked. It is common to sell a null result as the positive discovery of a new factor that modulates an established effect. However, it would often require additional experiments to make a convincing case. Another alternative is that the effect was never reliable under any conditions.”*

Reviewer #2: Makin, Rahman, & Bertamini (2020) revisit the question whether durations of symmetrical dot patterns are perceived as longer than those of asymmetrical dot patterns. Dot patterns, either random or containing 1, 2, 3, 4, or 5 symmetrical axes, were presented at varying durations (0.4, 0.6, 0.8 s, in addition to filler trials in which durations ranged from 0.1 to 0.9 s) which were subsequently estimated by 40 observers using a continuous scale from 0 to 1 s. Repeated-measures ANOVAs revealed no effects, neither of symmetry nor number of axes, thus failing to replicate previous effects. The authors argue that this null result is not a consequence of stimulus differences or low statistical power compared to previous studies.

To my mind, the described experiment is technically sound, even though the authors should include further methodological details to facilitate the readers’ understanding. The introduction features a long subsection about the relevance of null effects, yet the description of the relevant studies and their background is short and would – especially given this is a replication – benefit from relevant technical details. The same is valid for the discussion section: A more thorough examination of the differences between the studies in question and the current studies seems necessary, as the interpretation in terms of underlying mechanisms is not exactly obvious. In addition, a clarification on symmetry versus regularity should be included. The conducted analyses provide straightforward and sufficiently strong evidence for the null effect, but (unfortunately) no further attempts were made to scrutinize this result.

We agree with these points. We have expanded the introduction to focus more on the original rationale and expanded the discussion to examine possible explanations for the absent effect. We have also included more method details, as described below.

Major
(1) The manuscript is written in a way that suggests that previous findings could not be replicated, which may seem like a bold statement given that the studies in question have used (to some extent) different stimuli. Although I agree that to make a diagnostic inference about previous results a replication study need not be exactly similar to previous ones (e.g., Nosek & Errington, 2020), the possible constraints under which an effect might (only) be found, should be discussed. For instance, the most obvious candidate is stimulus complexity: Odgen et al. (2016) used 10x10 grids, as did Palumbo, Odgen et al. (2015a), whereas Palumbo et al. (2015b) used the same stimuli as in the present study, but not in a duration estimation task. Also, virtually all previous studies used longer durations up to 1.6 s, whereas here the longest duration was 0.9 s. This may be important in the context of Vierordt’s Law, suggesting a shorter intervals are more likely to be overestimated if longer reference intervals are present. In addition, could the scale have introduced a central tendency bias (e.g., Douven, 2017) instead of quantizing or ceiling effects? Moreover, what about stimulus size? Previous studies used 10x10 dva² patterns, that is at least twice the size of the stimuli used here (5 dva in diameter). I do not think that these differences invalidate the current study, but they should be both reported and discussed to provide an informative picture to the reader.

We have now been much clearer that this is a failed conceptual replication, not a failed replication. We have also considered how differences in the new experiment could have abolished the previously seen effect.

We have changed the title of the paper to avoid overselling as a failed pure replication:

*“No effect of multi-axis dot pattern symmetry on subjective duration.”*

We have also added an important clarification at the end of the abstract:

*“One possibility is that the effect of symmetry on subjective duration is limited to very specific experimental paradigms.”*

Most importantly, we have been more thorough in the discussion, considering possible explanations for inconsistency with previous research. Much of the following is new:

*“Our dot patterns were different from the checkerboards in Palumbo, Ogden et al. (2015) and Ogden et al. (2016) in a number of ways. For instance, they were more complex. Complexity can modulate perceived duration (Palumbo, Ogden, Makin, & Bertamini, 2014), so this could be part of the story. However, is unclear why increasing complexity should supress the difference between reflection and random conditions. Likewise, our stimuli were 5° diameter discs instead of 10° squares, but there is no obvious reason why this size change would abolish effect of symmetry on duration either.*

*Previous studies used durations ranging up to 1.6 seconds, while we used durations <1 second. Could this explain the inconsistencies with previous work? Ogden et al (2016) found the symmetry effect only for durations of less than a second, so it may be necessary to present a range of durations for the effect of symmetry to manifest at the lower end. Indeed, range effects are well known in the timing literature: Vierordt’s law states duration estimates tend to cluster round the mean presented (so the shorter durations are overestimated, and longer durations underestimated). Vierordt’s law could perhaps contribute to a multifaceted explanation for our null result.*

*A failed conceptual replication could also be uninteresting if the task had changed in an obviously problematic way. We cannot identify an obvious task-based explanation either. Palumbo, Ogden et al. (2015) and Ogden et al. (2016) used a verbal estimation task, where participants entered judgements using the keyboard. In contrast, our participants entered judgments using the mouse and continuous scale. Our new response entry method may have reduced ‘quantizing’ (where participants tend to give round number estimates such as “500 ms” or “1000 ms” Wearden, 2015). The extra precision could theoretically introduce ceiling effects that mask the effect of symmetry. However, the effect of objective duration on subjective duration was comparable to previous work (Current study η_p_^2^ = 0.85, Ogden et al. 2016, η_p_^2^ = 0.88, Palumbo, Ogden et al. 2015, η_p_^2^ = 0.76). This suggests that suppressive ceiling effects were no greater in the current experiment.*

*The above paragraphs demonstrate how failed conceptual replications can arise from previously unknown boundary conditions. Some change in stimulus or task, originally assumed to be trivial, has the unforeseen consequence of abolishing the expected effect. Palumbo, Ogden et al. (2015) and Ogden et al. (2016) might have been lucky by choosing parameters that worked. It is common to sell a null result as the positive discovery of a new factor that modulates an established effect. However, it would often require additional experiments to make a convincing case. Another alternative is that the effect was never reliable under any conditions.”*

(2) Curiously, the manuscript includes the study of Sasaki & Yamada (2017), which uses an stimulus and technique quite different from what was used by the authors’ and their previous studies. In this context, I came across a response by Sasaki & Yamada (2020) to this manuscript, which should be incorporated in a revised version. Like these authors, I share the concern that the underlying mechanisms that give rise to overestimation of duration of symmetrical or regular stimuli, respectively, might be quite different: On the one hand, valence and arousal may play a role, on the other hand, a neural energy account involving second-order orientation processing may contribute to an explanation of the results. Clearly, these two accounts are not mutually exclusive, but they should be discussed thoroughly to avoid misconceptions.

It is true that Sasaki and Yamada (2017) responded to our pre-print. We have now avoided the claim that our study is a simple failure to replicate Sasaki and Yamada (2017) in the abstract and introduction.

However, we do think our result is relevant to their conclusions, so we have mentioned Sasaki and Yamada (2017) in the discussion in a more careful way:

*“An effect of regularity on subjective duration was also reported by Sasaki and Yamada (2017). They found that more regular grid patterns appeared longer. We were pleased when these authors responded to a pre-print of this article on bioRxiv (Makin, Rahman, & Bertamini, 2020). They argued that our reflectional symmetry is not equivalent to their grid regularity, and that our failed replication does not reduce confidence their conclusions (Sasaki & Yamada, 2020). We are now agnostic. A new series of experiments may be the only way to determine which non-accidental arrangements reliably elongate subjective duration. Such a research program may confirm the dissociation between reflectional symmetry and grid regularity, proposed by Sasaki and Yamada (2020).”*

(3) Methodological details should be clarified or added to the manuscript to fulfil the rigor criterion. To just list a few: Were trials presented in a randomly interleaved fashion or blocked? The term N axis is unclear, but I assume it relates to the number of folds. If the SD of the number of dots changes with folds, was that the same for the random stimuli? How were folds included in the random condition? Were the stimulus controlled for luminance, surface area, (or incidental orientations), as in Palumbo, Odgen et al. (2015)? Would there be a possibility to control for these covariates post-hoc, as in a reverse correlation analysis?

We agree that more methodological details are required. Unfortunately, we cannot do reverse correlation analysis because all participants received different stimuli, and we do not have an exact record of those stimuli. However, we expanded on this in the stimulus section:

*“The stimulus construction algorithm is described in Makin et al. (2016) and Palumbo, Makin and Bertamini (2015). The circular frame was approximately 5 degrees in diameter, as in Makin et al. (2016). The patterns were generated by filling an implicit grid within pie-slice segments of the circle. The folds produced seams between the segments and local orientation cues, so it was essential to included 1-5 folds in the random condition as well, although these are not reflectional axes in the same sense (Figure 1 top row). Each cell could be occupied with a black dot, and the probability of occupation was set as at 0.4. This meant 40% of cells were occupied with a dot. The average number of dots (N-dots) was 525, although this mean varied with folds [1F = 514, 2F = 528, 3F = 521, 4F = 531, 5F = 532]. More importantly, there was some variability around the mean. SD of N-Dots changed with number of folds and was higher in for reflection [1F = 24, 2F = 37, 3F = 44, 4F = 57, 5F = 49] than random [1F = 14, 2F = 15, 3F = 17, 4F = 21, 5F = 17]). Although N-dots (and thus luminance) was more variable in the symmetry conditions, average N-dots (and thus average luminance) was the same in symmetry and random conditions.”*

Furthermore, due to the random nature of filler trials, were their physical durations on average similar? To account for varying durations in these trials, the reported durations could be normalized by their corresponding physical durations, which would also give a more interpretable estimate on the amount of underestimation. The factor physical duration was included in the rmANOVA as a continuous/metric one, do the results change if it is included as a discrete/ordinal one?

We have now corrected a few sections about the filler trials and done further analysis. Their mean objective duration was 0.6, exactly like the experimental trials (min 0.2, max = 1.0). (We previously misreported this as 0.1 and 0.9). We have also expanded on filler trial analysis in the method section:

“On filler trials, we also measure slope and intercept of the relationship between objective and subjective duration, as well as the correlation between objective and subjective duration. Although each participant was presented with a slightly different distribution of filler trial durations, these three metrics would be identical for ideal observers who make veridical duration estimates (intercept 0, slope 1, r = 1). We can thus use these metrics to assess whether performance on filler trials is influenced by regularity.”

Participants underestimated duration on filler trials. We have now included this explicitly in the results section:

*“Durations were again underestimated in filler trials, (mean estimate 0.461 seconds instead of the veridical 0.6 seconds, Figure 2B).”*

We can neatly capture responses to the filler trials by computing the slope and intercept of the relationships between objective and subjective duration for each participant. Figure 2 has now been expanded accordingly:

**Figure 2 Results.** A) Mean subjective duration in experimental trials. This is separated into reflection and random conditions with 1-5 folds. B) Mean subjective duration in filler trials in reflection and random conditions with 1-5 folds. C) Mean correlation of the relationship between objective and subjective duration in the filler trials. D) Intercepts of the relationship between objective and subjective duration in the filler trials. E) Slopes of the relationship between objective and subjective duration in the filler trials. Error bars = +/- 1 S.E.M.

This is now included in the results:

*“Finally, we calculated intercepts and slopes of this relationship. These metrics were also unaffected by regularity (Figure 2D and E, t (39) = -0.479, p = 0.634; t (39) = 0.674, p = 0.505).”*

We are not 100% clear what the recommendations are regarding rmANOVA? Our Duration factor had three discrete levels. For greater generality, we have now added the linear contrast, which treats the Duration factor as continuous.

Minor

“Therefore, there was no evidence that visual properties of the patterns altered performance” (p. 9): How do population-level correlations between actual and perceived duration indicate whether low-level visual features have an impact on duration estimation? This should be rephrased or elaborated on.

We have removed this during rewriting this section

“However, the symmetry was more salient here, and if anything, the effect of symmetry on duration should have been larger” (p. 10): Could the authors elaborate on why this should be the case? First, it is not instantly evident why symmetry should be more salient, given the higher complexity of the stimulus, and, second, is there other evidence that symmetry – varied parametrically – impacts subjective duration specifically?

We have removed this section. Elaboration leads to more confusion.

We do not know of any evidence beyond that in Sasaki and Yamada (2017), who included low, moderate and high regularity.

List of typos or grammatical issues:
- Sasaki & Yamada instead of “Sakaki & Yamada” (p. 5)
- “was more likely abolish the expected effect” (p. 10)
- “For instance null result might be…” (p. 10)
- “well included there were 1-5 folds in the random conditions” (p. 7)
- “Participants sat darkened cubicle” (p. 7)
- “duration estimate in was obtained in all 30 conditions” (p. 8)

Thanks
